# Responsiveness and minimal clinically important difference of SGRQ-I and K-BILD in idiopathic pulmonary fibrosis

**DOI:** 10.1186/s12931-020-01359-3

**Published:** 2020-04-21

**Authors:** Thomas Skovhus Prior, Nils Hoyer, Ole Hilberg, Saher Burhan Shaker, Jesper Rømhild Davidsen, Elisabeth Bendstrup

**Affiliations:** 1 Center for Rare Lung Diseases, Department of Respiratory Diseases and Allergy, Aarhus University Hospital, Palle Juul-Jensens Boulevard 99, DK-8200 Aarhus N, Denmark; 2 Department of Respiratory Medicine, Herlev and Gentofte Hospital, Copenhagen, Denmark; 3 Department of Respiratory Medicine, Vejle Hospital, Vejle, Denmark; 4 Department of Respiratory Medicine, Odense University Hospital, Odense, Denmark

**Keywords:** Idiopathic pulmonary fibrosis, IPF, Health-related quality of life, IPF-specific version of St. Georges respiratory questionnaire, SGRQ-I, King’s Brief Interstitial Lung Disease questionnaire, K-BILD, minimal clinically important difference, Responsiveness, Longitudinal validity

## Abstract

**Background:**

Idiopathic pulmonary fibrosis (IPF) specific version of St. George’s Respiratory Questionnaire (SGRQ-I) and King’s Brief Interstitial Lung Disease questionnaire (K-BILD) are validated health-related quality of life (HRQL) instruments, but no or limited data exist on their responsiveness and minimal clinically important difference (MCID). The objectives of this study were to assess responsiveness of SGRQ-I and K-BILD and determine MCID separately for deterioration and improvement in a large, prospective cohort of patients with IPF in a real-world setting.

**Methods:**

Consecutive patients with IPF were recruited. SGRQ-I, K-BILD, SGRQ, Shortness of Breath Questionnaire, pulmonary function tests and 6-min walk test measurements were obtained at baseline and at six and 12 months; at six and 12 months, patients also completed Global Rating of Change Scales. Responsiveness was assessed using correlation coefficients and linear regression. Cox regression was used for mortality analyses. MCID was estimated using receiver operating characteristic curves with separate analyses for improvement and deterioration.

**Results:**

A total of 150 IPF patients were included and 124 completed the 12-month follow-up. Based on all HRQL anchors and most physiological anchors, responsiveness analyses supported the evidence pointing towards SGRQ-I and K-BILD as responsive instruments. Multivariate analyses showed an association between SGRQ-I and mortality (HR: 1.18, 95% CI: 1.02 to 1.36, *p* = 0.03) and a trend was found for K-BILD (HR: 0.82, 95% CI: 0.64 to 1.05, *p* = 0.12). MCID was estimated for all domains of SGRQ-I and K-BILD. MCID for improvement differed from deterioration for both SGRQ-I Total (3.9 and 4.9) and K-BILD Total (4.7 and 2.7).

**Conclusions:**

SGRQ-I and K-BILD were responsive to change concerning both HRQL and most physiological anchors. MCID was determined separately for improvement and deterioration, resulting in different estimates; especially a smaller estimate for deterioration compared to improvement in K-BILD.

**Trial registration:**

Clinicaltrials.gov, no. NCT02818712. Registered 30 June 2016.

## Background

Idiopathic pulmonary fibrosis (IPF) is the most burdensome interstitial lung disease (ILD). It is a chronic, fibrotic lung disease characterised by progressive decline in lung function and increasing dyspnoea [1]. Cough, fatigue, loss of emotional well-being and social isolation are other consequences of the disease [2]. Along with a wide range of comorbidities, patients with IPF often experience impaired health-related quality of life (HRQL) [2, 3]. As the disease progresses, the symptom burden increases resulting in decreasing HRQL, and in the terminal phase of the disease, HRQL plummets considerably [4]. Antifibrotic treatments successfully slow down lung function decline, but do not improve HRQL convincingly [5, 6].

Patient-reported outcome measures (PROMs) are used to quantify HRQL. Like any other measurement instrument, PROMs must be tested to ensure sufficient validity and reliability. It is essential to evaluate an instrument’s ability to respond to change in health status (responsiveness), before it can be used as an endpoint in longitudinal studies. Another fundamental aspect is the minimal clinically important difference (MCID) denoting the smallest change in score of the instrument perceived as clinically relevant. Therefore, longitudinal studies are needed to ensure valid and responsive instruments in the target population studied.

A modified version of Saint George’s Respiratory Questionnaire (SGRQ) was developed for patients with IPF (SGRQ-I) [7, 8]. To our knowledge, no studies have examined responsiveness or MCID of SGRQ-I, and assessment of the longitudinal validity of SGRQ-I is important before implementing the instrument in clinical trials or daily practice.

King’s Brief Interstitial Lung Disease questionnaire (K-BILD) was developed as a HRQL instrument for patients with ILD [9] and has recently been validated in IPF [10]. Even though K-BILD is used in clinical trials [11, 12], responsiveness and MCID have not yet been sufficiently determined [13, 14]. Validation of an instrument is an iterative process increasing robustness by evaluation in different cohorts.

Although fibrotic changes in IPF are irreversible often resulting in decreased HRQL and pulmonary function, improvement in HRQL is seen in some patients [14–16]. MCID should thus be examined separately, as estimates may differ [17].

The aim of this study was to assess the responsiveness of SGRQ-I and K-BILD and determine MCID separately for deterioration and improvement in both instruments in a large prospective cohort of patients with IPF in a real-world setting.

## Materials and methods

### Study subjects

Consecutive patients with IPF were recruited from outpatient clinics at the three tertiary ILD centres in Denmark from August 2016 to March 2019. To increase generalisability, both incident and prevalent patients with IPF were enrolled. Adult patients > 18 years diagnosed with IPF in accordance with international guidelines were eligible for inclusion [18, 19]. Exclusion was based on linguistic or intellectual barriers preventing completion of the questionnaires. Other studies on K-BILD and SGRQ-I have been based on the same cohort of patients with IPF [8, 10].

All patients gave written informed consent. The study was approved by the Central Denmark Region Committee on Health Research Ethics (case no. 1–10–72-87-16). The study was registered at clinicaltrials.gov (NCT02818712) before initiation.

### Methods

All patients completed SGRQ-I, K-BILD, SGRQ and University of California San Diego Shortness of Breath questionnaire (SOBQ) at baseline and at six and 12 months; at six and 12 months, patients also completed Global Rating of Change Scales (GRCS). Assessment of forced vital capacity (FVC), diffusing capacity of the lung for carbon monoxide (DLCO) and a 6-min walk test were performed at all three time points. These measurements were used as anchors for both responsiveness and MCID analyses.

*SGRQ-I* is an IPF-specific version of SGRQ measuring HRQL by 34 self-completed items [7]. Various response options are used when completing the instrument, and results are reported as total score and three domain scores: Impacts, Activities and Symptoms; lower scores indicate better HRQL.

*K-BILD* is a self-completed HRQL questionnaire developed for patients with ILDs [9]. It is composed of 15 items scored on a 7-point Likert scale. Results are reported as a total score and three domain scores: Psychological, Breathlessness and activities and Chest symptoms. Logit-transformed scores range from 0 to 100, and higher scores reflect higher HRQL.

*SGRQ* comprises 50 items in a self-completed HRQL questionnaire, which has been validated in IPF including studies on responsiveness [20, 21]. Scoring is similar to SGRQ-I, and MCID of five points has been reported in IPF [22]. Based on these estimates, patients with a change in SGRQ below five points were regarded as unchanged, whereas a change equal to or larger than ±5 points were regarded as improved or deteriorated, respectively.

*GRCS* are self-completed 11-point Likert scales developed to evaluate changes in lung-related health status of patients between visits [23]. The numeric response scales range from − 5 (Very much worse) over 0 (Unchanged) to 5 (Very much better). Different GRCS were designed to encompass the overall lung-related health status and the three domains of SGRQ-I and K-BILD. The score of each GRCS was classified as deteriorated (− 5 to − 2), unchanged (− 1 to 1) or improved (2 to 5).

*SOBQ* measures dyspnoea related to daily activities in a 24-item self-completed questionnaire [24]. Responses are registered on a 6-point scale, and total score ranges from 0 to 120; lower scores indicate less dyspnoea. SOBQ has been validated for use in IPF and has shown to be responsive to changes over time [16]. Patients were divided into deteriorated (ΔSOBQ ≤ − 8), unchanged (ΔSOBQ − 8 to 8) or improved (ΔSOBQ ≥8), in concordance with the MCID of SOBQ in IPF [16].

Permission to use the instruments was obtained and all instruments were completed in the original format on paper.

Both *FVC* and *DLCO* are widely used in IPF as measures of disease severity, and the distance walked during the 6-min walk test (6MWD) is used as an assessment of functional capacity. All three tests are predictive of survival in patients with IPF [19, 25]. Absolute changes in FVC % predicted from baseline to 12 months were divided into deteriorated (ΔFVC ≤ − 6%), unchanged (ΔFVC − 6 to 6%) and improved (ΔFVC ≥6%) in accordance with estimates of MCID for FVC in ILD [26, 27]. Absolute changes in DLCO % predicted from baseline to 12 months were also categorised into deteriorated (ΔDLCO ≤ − 10%), unchanged (ΔDLCO − 10 to 10%) and improved (ΔDLCO ≥10%), based on the intraindividual variability in DLCO measurements, as no MCID has been reported for IPF [28]. Likewise, 6MWD was divided into deteriorated (Δ6MWD ≤ − 28 m), unchanged (Δ6MWD − 28 m to 28 m) and improved (Δ6MWD ≥ 28 m), based on MCID estimates for 6MWD in IPF [29].

### Statistical analysis

Frequency tables, mean (standard deviation, SD) or median (interquartile range, IQR) were used for descriptive data. Questionnaires missing > 15% answers or missing domain/total scores were excluded from the analyses. *P*-values < 0.05 were considered statistically significant. Data were analysed using STATA 14.2 (StataCorp, College Station, Texas).

#### Responsiveness

A mixed effects model with random intercept and cluster effect for centre (using the “Clustered Sandwich Estimator”) to take the possible within centre correlation into account was used to analyse changes in HRQL over 12 months.

The association between changes in SGRQ-I and K-BILD and changes in anchors (GRCS, SOBQ, SGRQ, FVC % predicted, DLCO % predicted, 6MWD) from baseline to 12 months were assessed by Pearson’s correlation coefficients. Due to inverse scoring algorithms, negative correlations were expected for SGRQ-I: GRCS, FVC, DLCO and 6MWD and for K-BILD: SGRQ and SOBQ.

Furthermore, patients were divided into three equally large groups according to stage of disease by SOBQ, SGRQ, FVC, DLCO and 6MWD. For GRCS, patients were divided into deteriorated, unchanged or improved. Changes in SGRQ-I and K-BILD from baseline to 12 months in the groups were compared by linear regression to assess the linear relationship between changes in disease severity and changes in SGRQ-I and K-BILD.

Cox regression analyses were used to examine the association between SGRQ-I and K-BILD baseline score and mortality during the 12-month follow-up. Age and FVC % predicted were included as covariates in the model.

#### MCID analyses

Receiver operating characteristic (ROC) curves were used to estimate MCID of SGRQ-I and K-BILD. A combination of anchor-based and distribution-based methods is recommended to determine MCID [17, 30], and ROC curves incorporate both approaches [31]. As anchors should be related to the instrument investigated [30], only anchors with a prespecified correlation > 0.3 were included in the analyses. Patients were categorised as improved, unchanged or deteriorated according to the thresholds (mainly MCIDs) of the anchors as described above. MCID for SGRQ-I and K-BILD was analysed by separate ROC curves for deterioration (unchanged vs. deteriorated patients) and improvement (unchanged vs. improved patients) according to the anchors [31]. The MCID estimate for SGRQ-I and K-BILD was the optimal cut-off point of the ROC curves with equal sensitivity and specificity. Sensitivity analyses were performed to assess whether baseline HRQL had an influence on MCID estimates. Patients were divided into two groups according to baseline SGRQ-I or K-BILD score; MCID estimates were calculated using ROC curves as described above in the 50% of patients with the best HRQL and afterwards in the 50% with the lowest HRQL.

## Results

A total of 150 patients with IPF were included at baseline (Table 1). The majority were males with a history of smoking and most patients received antifibrotic treatment. At baseline, FVC was well preserved while DLCO was moderately impaired. A total of 124 patients (83%) completed the 12-month follow-up. Causes of withdrawal from the study were: Death (*n* = 16), could not complete questionnaires (n = 1), could not attend outpatient visit (*n* = 3), patient’s wish to withdraw (*n* = 6).

**Table 1:** Baseline demographics of the patients (*n* = 150) [8, 10]

Male, n (%)	122 (81.3%)
Age (years), mean (SD)	72.9 (6.2)
Months since diagnosis, median (IQR)	6 (0–21)
Smoking status	
Current, n (%)	9 (6.0%)
Former, n (%)	101 (67.3%)
Never, n (%)	40 (26.6%)
Long-term oxygen therapy, n (%)	19 (12.7%)
Antifibrotic treatment, n (%)^a^	85 (56.7%)
FVC (% predicted), mean (SD)	87.2 (23.1)
DLCO (% predicted), mean (SD)	48.4 (14.1)
6MWD (m), mean (SD)	450.3 (112.5)
K-BILD total, mean (SD)	58.3 (12.4)
SGRQ-I total, mean (SD)	42.9 (22.3)
SOBQ, mean (SD)	34.6 (25.3)
SGRQ, mean (SD)	40.8 (19.4)

^a^: 1/3 of the patients was incident and hence, did not receive antifibrotic treatment at baseline. *SD* Standard deviation, *IQR* Interquartile range, *FVC* Forced vital capacity, *DLCO* diffusion capacity of the lung for carbon monoxide, *6MWD* distance walked during the 6-min walk test, *K-BILD* King’s Brief Interstitial Lung Disease questionnaire, *SGRQ-I* IPF-specific version of St. George’s Respiratory Questionnaire, *SOBQ* University of California, San Diego Shortness of Breath Questionnaire, *SGRQ* St. George’s Respiratory Questionnaire

### Responsiveness

**Fig. 1:**
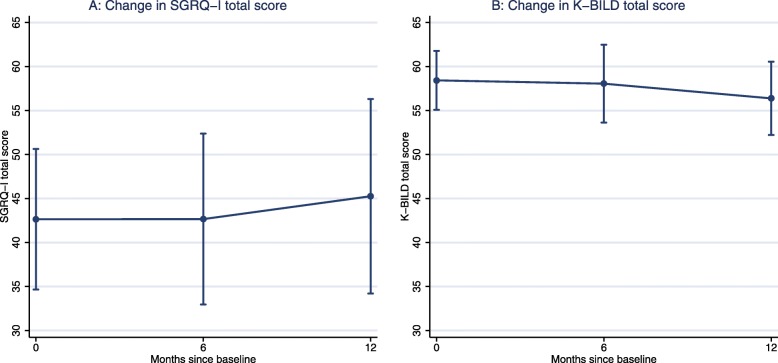
SGRQ-I (A) and K-BILD (B) Total score at baseline, six months and 12 months. Dots indicate mean scores and whiskers illustrate 95% confidence intervals. *SGRQ-I*: IPF-specific version of the Saint George’s Respiratory Questionnaire, *K-BILD*: King’s Brief Interstitial Lung Disease questionnaire

A trend towards deterioration of HRQL was observed from baseline to 12 months (SGRQ-I: 2.61 (95% CI: − 0.82 to 6.04, *p* = 0.14), K-BILD: -2.05 (95% CI: − 4.15 to 0.05), *p* = 0.06); the largest difference occurred between six and 12 months (SGRQ-I: 2.59 (95% CI: 1.20 to 3.99, *p* < 0.001), K-BILD: -1.67 (95% CI: − 2.08 to − 1.26), p < 0.001) (Fig. 1). Improvement in the physiological anchors was observed in 2–19% of patients and in 36–41% of patients in the HRQL anchors, while deterioration was observed in physiological anchors in 16–23% and in HRQL anchors in 15–40% of patients.

Correlations between changes in SGRQ-I and K-BILD and changes in anchors from baseline to 12 months are shown in Table 2. The direction of all correlations was as expected. Correlations for patients receiving antifibrotic treatment at baseline were similar.

**Table 2:** Correlations between changes in SGRQ-I or K-BILD domains and changes in anchors from baseline to 12 months

	GRCS	ΔSOBQ	ΔSGRQ	ΔFVC%	ΔDLCO%	Δ6MWD
**ΔSGRQ-I Total**	−0.57	0.60	0.94	−0.21	−0.17	− 0.49
**ΔSGRQ-I Symptoms**	−0.44	0.39	0.54	−0.25	−0.05	− 0.33
**ΔSGRQ-I Activities**	−0.47	0.44	0.68	−0.21	−0.18	− 0.29
**ΔSGRQ-I Impacts**	−0.46	0.53	0.87	−0.09	−0.15	− 0.47
**ΔK-BILD Total**	0.49	−0.46	−0.63	0.20	0.19	0.28
**ΔK-BILD Psychological**	0.44	−0.38	−0.56	0.17	0.09	0.31
**ΔK-BILD Breathlessness and activities**	0.38	−0.47	−0.63	0.19	0.30	0.31
**ΔK-BILD Chest symptoms**	0.33	−0.26	−0.42	0.18	0.11	0.12

Δ: Change from baseline to 12 months; *SGRQ-I* IPF-specific version of the Saint George’s Respiratory Questionnaire, *K-BILD* King’s Brief Interstitial Lung Disease questionnaire, *CI* Confidence interval, *GRCS* Global rating of change scales, *SOBQ* University of California San Diego Shortness of Breath questionnaire, *SGRQ* Saint George’s Respiratory Questionnaire, *FVC%* Forced vital capacity % predicted, *DLCO%* Diffusing capacity of the lung for carbon monoxide % predicted, *6MWD* Distance walked during the 6-min walk test

**Fig. 2:**
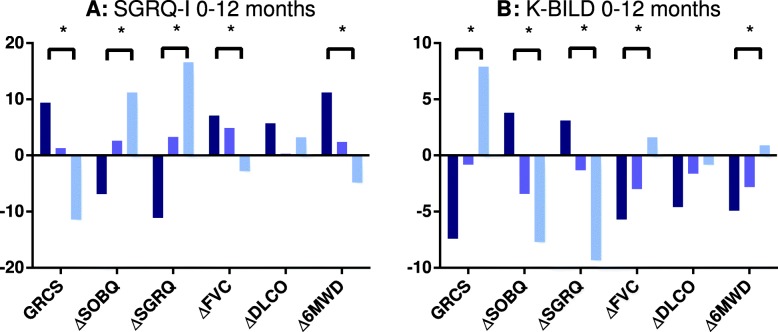
Results of linear regression comparing SGRQ-I (A) and K-BILD (B) scores from baseline to 12 months in groups of different stages of disease. Patients were divided into three equally large groups according to stage of disease by SOBQ, SGRQ, FVC, DLCO and 6MWD. For GRCS, patients were divided into deteriorated, unchanged or improved (see Additional file 1 for group details). *: *p* < 0.05 for linear effect. *Δ*: Change from baseline to 12 months; *SGRQ-I*: IPF-specific version of the Saint George’s Respiratory Questionnaire, *K-BILD*: King’s Brief Interstitial Lung Disease questionnaire, *CI*: Confidence interval, *GRCS*: Global Rating of Change Scales, *SOBQ*: University of California San Diego Shortness of Breath questionnaire, *SGRQ*: Saint George’s Respiratory Questionnaire, *FVC*: Forced vital capacity, *DLCO*: Diffusing capacity of the lung for carbon monoxide, *6MWD*: Distance walked during the 6-min walk test

The association between changes in SGRQ-I and K-BILD and changes in anchors from baseline to 12 months are shown in Fig. 2 (see Additional file 1 for details). All changes were in the expected direction.

In the univariate analyses, low baseline HRQL measured in 5-point intervals was associated to increased mortality during follow-up for both SGRQ-I (HR: 1.28, 95% CI: 1.12 to 1.46, *p* < 0.001) and K-BILD (HR: 0.68, 95% CI: 0.54 to 0.84, *p* = 0.001). After adjustment for age and FVC % predicted, results remained significant for SGRQ-I (HR: 1.18, 95% CI: 1.02 to 1.36, *p* = 0.03) and a trend was found for K-BILD (HR: 0.82, 95% CI: 0.64 to 1.05, *p* = 0.12). Please refer to Additional File 2 for results of 1-point intervals.

### Minimal clinically important difference

Table 3 presents the estimates of MCID for SGRQ-I and K-BILD for improvement and deterioration separately. Sensitivity analyses of MCID estimates for SGRQ-I and K-BILD total scores in the 50% of patients with the best HRQL (SGRQ-I improvement 3.3, deterioration 5.4; K-BILD improvement 2.0, deterioration 4.4) and the 50% with the worst HRQL (SGRQ-I improvement 5.8, deterioration 4.4; K-BILD improvement 5.0, deterioration 3.7) were similar. MCID estimates for patients receiving antifibrotic treatment at baseline (SGRQ-I improvement 6.1, deterioration 5.7; K-BILD improvement 4.7, deterioration 2.3) were also comparable.

**Table 3:** Mean and range of MCID estimates for SGRQ-I and K-BILD domains based on change in anchors from baseline to 12 months

Domains	Improvement		Deterioration	
	*Mean*	*Range*	*Mean*	*Range*
SGRQ-I total	3.9	0.7–5.5	4.9	1.3–7.6
SGRQ-I Symptoms	9.0	7.3–13.5	8.1	7.2–10.3
SGRQ-I Activities	9.8	9.6–10.0	10.4	9.6–10.9
SGRQ-I Impacts	5.4	2.1–8.8	5.4	4.0–8.4
K-BILD Total	4.7	2.0–5.0	2.7	2.0–3.0
K-BILD Psychological	4.8	2.0–6.0	3.5	1.0–7.0
K-BILD Breathlessness and activities	3.6	0.0–6.0	3.6	2.0–6.0
K-BILD Chest symptoms	7.0	4.0–10.0	6.0	3.0–9.0

*MCID* Minimal clinically important difference, *SGRQ-I* IPF-specific version of the Saint George’s Respiratory Questionnaire, *K-BILD* King’s Brief Interstitial Lung Disease questionnaire

## Discussion

This is the first study prospectively examining responsiveness and MCID in a large sample of patients with IPF in a real-world, multicentre setting. Responsiveness was investigated using different approaches and MCID was determined separately for improvement and deterioration. Sensitivity analyses of patients with different baseline HRQL were performed. Results indicated that SGRQ-I and K-BILD are responsive to change according to all HRQL anchors and most physiological anchors. Estimates of MCID total scores differed by 1–2 points between improvement and deterioration. Results were comparable in the sensitivity analyses. An association between SGRQ-I and mortality was observed and a trend was found between K-BILD and mortality.

The ability of an instrument to respond to changes is essential to longitudinal validity; otherwise assessment of MCID is irrelevant. Hence, responsiveness should be assessed by different methods. In this study, we used both correlation analyses and compared groups with different disease stages by linear regression. Both methods indicated that SGRQ-I and K-BILD responded to changes in all HRQL and most physiological anchors; scores of the two instruments changed in concordance with changes in the anchors. The weaker correlations to physiological than to HRQL anchors were expected, as cross-sectional studies of K-BILD and SGRQ-I have shown similar results [7–10]. As a result of measurement error on two measures, correlations between changes in scores are expected to be smaller. This may explain the generally weaker correlations in this longitudinal study compared to the cross-sectional studies on SGRQ-I and K-BILD [7–10]. DLCO showed the weakest associations to SGRQ-I and K-BILD in both analyses. Correspondingly, significant changes in another study using DLCO as an anchor were not achieved [16]. One explanation might be the considerable inherent variability in measurements of DLCO and thus less significant results [28]. All things considered, the analyses supported the evidence pointing towards SGRQ-I and K-BILD as responsive instruments.

Responsiveness of SGRQ-I has not been assessed previously, but it has been evaluated for SGRQ [22, 32]. Responsiveness of K-BILD was only briefly described by Sinha et al. [13], whereas Nolan et al. limited their investigations to correlation analyses [14]. Interestingly, correlations were stronger to SOBQ (SGRQ-I: 0.39 to 0.60; K-BILD: − 0.47 to − 0.26;) than to Medical Research Council dyspnoea score (SGRQ: 0.25 to 0.39; K-BILD: − 0.29 to − 0.23) and Transition Dyspnoea Index (SGRQ: − 0.47 to − 0.28) [14, 22, 32]. Correlations between K-BILD and SGRQ were also stronger (− 0.63 to − 0.42) than to the Chronic Respiratory Questionnaire (0.27 to 0.54) [14]. These divergencies may be explained by differences between the psychometric properties of the instruments and it is thus important to choose anchors with established responsiveness [30]. Both SOBQ and SGRQ have been longitudinally validated for use in IPF [15, 16]. Chronic Respiratory Questionnaire has only been validated for longitudinal use in chronic obstructive pulmonary disease [33], and neither Medical Research Council dyspnoea score nor Transition Dyspnoea Index have, to our knowledge, been validated for longitudinal use in IPF. The association between low HRQL and increased mortality has been investigated in other studies using for instance SGRQ and SOBQ. In concordance with the IPF-specific SGRQ-I, both baseline SGRQ score and changes in SGRQ and SOBQ scores were found to be prognostic factors in patients with IPF [4, 34]. The small number of mortalities and less advanced disease in our cohort might explain why only a trend was observed between K-BILD and mortality.

Currently, there is no consensus on the best method to estimate MCID. Anchor-based and distribution-based methods are used, and both have strengths and limitations [35]. Anchor-based methods use an external measurement as an anchor with a well-determined threshold for improvement or deterioration. The advantage is that the definition of a ‘minimal clinically important’ difference is well described and included in the method. On the other hand, the variability of the measurements is not taken into account. Distribution-based methods incorporate the variability by comparing change in the PROM to a measure of variation, hence obtaining a more standardised result. The disadvantage is that there is no good definition of a ‘clinically important’ change. These measures may also be different when comparing a homogeneous and a heterogeneous group. A combination of the methods to estimate MCID was proposed [17, 30] and therefore, we used the ROC curve approach proposed by de Vet et al., as this method combines anchor- and distribution-based methods [31].

This study is the first to assess MCID for SGRQ-I. MCID for SGRQ was determined in two studies based on patients with IPF from clinical trials [15, 22]. MCID for K-BILD has been determined in two other studies. Sinha et al. estimated a combined MCID in a mixed group of ILDs without specific analyses for IPF, probably due to a small sample size [13]. Nolan et al. determined MCID after an intervention of pulmonary rehabilitation [14]. MCIDs were marginally higher in the mentioned studies compared to our results: SGRQ vs. SGRQ-I Total (4.0–6.6 vs. 3.9–4.9) [15, 22] and K-BILD Total (3.9–5.0 vs. 2.7–4.7, [13, 14]. There may be numerous reasons explaining the differences between the studies. The larger sample size in our study increases the statistical power to determine a more exact estimate of MCID (our study *n* = 150, Nolan et al. *n* = 105, Sinha et al. *n* = 57 (17 IPF)). Differences in the composition of the cohorts with regard to age, gender and disease severity may all affect results. Additionally, different time frames, pulmonary rehabilitation vs. no intervention and variation in methods and anchors were used. The other studies used distribution-based approaches which may explain the larger estimates [36]. Also, the generalisability of the results of the other studies is limited due to the mixed group of ILDs and selection of patients for pulmonary rehabilitation or clinical trials.

Most studies only determined a single MCID for both improvement and deterioration. Even though HRQL generally deteriorated, up to one third of the patients experienced improvement in the anchors, and MCID for this group of patients should thus be analysed separately. As our study shows, MCID is different concerning improvement and deterioration, respectively; the largest difference was observed in SGRQ-I Total and Symptoms along with K-BILD Total and Psychological domains. Evidence of different MCIDs for improvement and deterioration has also been reported in other diseases [15, 36]. Hence, changes in SGRQ-I and K-BILD scores should be interpreted separately depending on the direction of change. Generally, the sensitivity analyses showed comparable results. The largest deviations were observed in MCID for improvement in SGRQ-I among patients with the best HRQL (5.8) and improvement in K-BILD among patients with the worst HRQL (2.0). This is consistent with a clinically important change having to be large in patients with good HRQL and smaller in patients with worse HRQL. Antifibrotic treatment at baseline hardly changed MCID estimates for K-BILD, whereas MCID estimates for SGRQ-I were slightly larger in this subgroup. However, a large proportion of the patients not receiving antifibrotic treatment at baseline (51%) initiated antifibrotic treatment during the 12-month follow-up, and these patients were included in the initial analyses.

Improvement in physiological and HRQL anchors observed in our study has also been reported in other IPF studies. In the INPULSIS trial, 19% of patients improved in FVC and up to 36% improved in HRQL anchors [15]. Comparable results have been reported in other studies [13, 16]. Improvement in HRQL may be due to better coping strategies for living with a severe disease, rehabilitation and oxygen treatment. The confidence intervals of change in SGRQ-I from baseline to 12 months are wider than the confidence intervals of K-BILD. An explanation might be the different response options in the two instruments; K-BILD uses the same Likert scale for all items, whereas SGRQ-I (and SGRQ) has a variety of response options throughout the instrument. This could lead to a larger variation in SGRQ-I scores as the instrument is less intuitive to complete. During the study, more patients needed guidance on how to complete SGRQ-I than K-BILD. As K-BILD is shorter, easier to complete and has comparable validity and reliability, we would recommend using K-BILD instead of SGRQ-I for future studies and in clinical practice.

This study had several strengths. First of all, a cohort of both incident and prevalent patients with IPF were recruited from multiple centres with very limited exclusion criteria, constituting a broad sample of the background IPF population, which enhances the external validity of our results. Secondly, MCID was determined separately for improvement and deterioration. The results revealed different estimates, displaying the importance of performing independent analyses for each direction of change. Furthermore, sensitivity analyses were performed to assess the robustness of the results. A limitation to our study was the recall bias associated with GRCS. It can be difficult to recall your health status 12 months back and compare it to your current health status. Still, GRCS can be easily interpreted, tailored to reflect specific domains of a PROM and have been reported to be reliable, valid and sensitive to change [23]. In addition, GRCS provide a simple assessment of the patients’ perception of their current HRQL.

## Conclusions

SGRQ-I and K-BILD were responsive to change in both HRQL and physiological anchors relevant to patients with IPF. MCID was determined separately for improvement and deterioration, resulting in different estimates; especially a smaller estimate for deterioration compared to improvement in K-BILD. Generalisability of the results was improved by our large cohort of unselected patients with IPF recruited from multiple centres. Our results can facilitate the use and interpretation of SGRQ-I and K-BILD in clinical practice and clinical trials incorporating HRQL outcomes.

## Supplementary information

**Additional file 1.** Results of linear regression comparing SGRQ-I or K-BILD Total scores in groups of different stages of disease from baseline to 12 months. Patients were divided into three equally large groups according to stage of disease by SOBQ, SGRQ, FVC, DLCO and 6MWD. For GRCS, patients were divided into deteriorated, unchanged or improved.

**Additional file 2.** Cox regression analyses with 1-point intervals.

## Data Availability

The datasets collected and analysed during the current study are not publicly available due to information that could compromise research participant privacy, but are available from the corresponding author on reasonable request.

## Abbreviations

6MWD: Distance walked during the 6-min walk test
DLCO: Diffusing capacity of the lung for carbon monoxide
FVC: Forced vital capacity
GRCS: Global Rating of Change Scales
HRQL: Health-related quality of life
ILD: Interstitial lung disease
IPF: Idiopathic pulmonary fibrosis
K-BILD: The King’s Brief Interstitial Lung Disease questionnaire
MCID: Minimal clinically important difference
PROM: Patient-reported outcome measures
ROC: Receiver operating characteristic
SGRQ: The St. George’s Respiratory Questionnaire
SGRQ-I: The IPF-specific version of the St. George’s Respiratory Questionnaire
SOBQ: The University of California, San Diego Shortness of Breath Questionnaire
