# Supplementary material for: Responsiveness and minimal clinically important difference of SGRQ-I and K-BILD in idiopathic pulmonary fibrosis
Source: Respir Res. 2020 Apr 21;21:91. doi: 10.1186/s12931-020-01359-3 (PMC7175493; doi:10.1186/s12931-020-01359-3)
Supplement: Supplementary file 1 — Additional file 1. Results of linear regression comparing SGRQ-I or K-BILD Total scores in groups of different stages of disease from baseline to 12 months. Patients were divided into three equally large groups according to stage of disease by SOBQ, SGRQ, FVC, DLCO and 6MWD. For GRCS, patients were divided into deteriorated, unchanged or improved. [file 12931_2020_1359_MOESM1_ESM.docx]

**Additional file 1**

| **Anchor** | | ***Baseline to 12 months*** | | | |
| --- | --- | --- | --- | --- | --- |
|  |  | **ΔSGRQ-I Total (95% CI)** | | **ΔK-BILD Total** | |
|  |  | **Estimate (95% CI)** | **Overall linear effect** | **Estimate (95% CI)** | **Overall linear effect** |
| **GRCS** | Deteriorated (*n* = 46) | 9.4 (5.4 to 13.3) | <0.0001* | -7.4 (-10.3 to -4.5) | <0.0001* |
|  | Unchanged (*n* = 55) | 1.3 (-2.2 to 4.8) |  | -0.8 (-3.4 to 1.9) |  |
|  | Improved (*n* = 49) | -11.4 (-18.4 to -4.3) |  | 7.9 (2.9 to 13.0) |  |
| **ΔSOBQ** | Drop by 2 points or more (*n* = 34) | -6.9 (-11.3 to -2.6) | <0.0001* | 3.8 (0.5 to 7.2) | <0.0001* |
|  | Drop by 1 point – increase by 5 points (*n* = 38) | 2.6 (-1.5 to 6.7) |  | -3.4 (-6.6 to -0.2) |  |
|  | Increase by 10 points or more (*n* = 39) | 11.2 (7.3 to 15.2) |  | -7.7 (-10.8 to -4.5) |  |
| **ΔSGRQ** | Drop by 0.9 points or more (*n* = 39) | -11.1 (-14.0 to -8.2) | <0.0001* | 3.1 (-0.2 to 6.3) | <0.0001* |
|  | Drop by 0.8 points – increase by 8.1 points (*n* = 39) | 3.3 (0.3 to 6.2) |  | -1.3 (-4.6 to 2.0) |  |
|  | Increase by 8.1 points or more (*n* = 39) | 16.6 (13.7 to 19.5) |  | -9.3 (-12.6 to -6.1) |  |
| **ΔFVC** | Drop by 5% or more (*n* = 41) | 7.1 (2.5 to 11.6) | 0.002* | -5.7 (-9.1 to -2.3) | 0.003* |
|  | Drop by 4% - increase by 2% (*n* = 38) | 4.9 (0.2 to 9.5) |  | -3.0 (-6.5 to 0.5) |  |
|  | Increase by 3% or more (*n* = 44) | -2.8 (-7.2 to 1.6) |  | 1.6 (-1.8 to 5.0) |  |
| **ΔDLCO** | Drop by 5% or more (*n* = 37) | 5.7 (0.7 to 10.7) | 0.08 | -4.6 (-8.3 to -0.8) | 0.08 |
|  | Drop by 4% - increase by 0.6% (*n* = 43) | 0.3 (-4.4 to 5.0) |  | -1.6 (-5.1 to 1.8) |  |
|  | Increase by 0.7% or more (*n* = 42) | 3.2 (-1.4 to 7.7) |  | -0.8 (-4.3 to 2.7) |  |
| **Δ6MWD** | Drop by 29 m or more (*n* = 39) | 11.2 (6.8 to 15.6) | <0.0001* | -4.9 (-8.5 to -1.2) | 0.03* |
|  | Drop by 28 m – increase by 3 m  (*n* = 39) | 2.4 (-1.7 to 6.6) |  | -2.8 (-6.3 to 0.7) |  |
|  | Increase by 4 m or more (*n* = 39) | -4.8 (-9.1 to -0.6) |  | 0.9 (-2.7 to 4.5) |  |

Results of linear regression comparing SGRQ-I or K-BILD Total scores in groups of different stages of disease from baseline to 12 months. Patients were divided into three equally large groups according to stage of disease by SOBQ, SGRQ, FVC, DLCO and 6MWD. For GRCS, patients were divided into deteriorated, unchanged or improved.

*: p < 0.05 for linear effect. Δ: Change from baseline to 12 months; *SGRQ-I*: IPF-specific version of the Saint George’s Respiratory Questionnaire, *K-BILD*: King’s Brief Interstitial Lung Disease questionnaire, *CI*: Confidence interval, *GRCS*: Global Rating of Change Scales, *SOBQ*: University of California San Diego Shortness of Breath questionnaire, *SGRQ*: Saint George’s Respiratory Questionnaire, *FVC*: Forced vital capacity, *DLCO*: Diffusing capacity of the lung for carbon monoxide, *6MWD*: Distance walked during the 6-minute walk test
