# Supplementary material for: Responsiveness and minimal clinically important difference of SGRQ-I and K-BILD in idiopathic pulmonary fibrosis
Source: Respir Res. 2020 Apr 21;21:91. doi: 10.1186/s12931-020-01359-3 (PMC7175493; doi:10.1186/s12931-020-01359-3)
Supplement: Supplementary file 2 — Additional file 2. Cox regression analyses with 1-point intervals. [file 12931_2020_1359_MOESM2_ESM.docx]

**Additional file 2**

|  | **Hazard ratio** | **95% CI** | **p-value** |
| --- | --- | --- | --- |
| **SGRQ-I** |  |  |  |
| Unadjusted | 1.05 | 1.02 to 1.08 | p<0.001 |
| Adjusted FVC + age | 1.03 | 1.00 to 1.07 | p=0.02 |
|  |  |  |  |
| **K-BILD** |  |  |  |
| Unadjusted | 0.92 | 0.88 to 0.96 | p<0.001 |
| Adjusted FVC + age | 0.96 | 0.91 to 1.01 | p=0.09 |

Cox regression analyses with 1-point intervals*.*

*SGRQ-I*: IPF-specific version of the Saint George’s Respiratory Questionnaire, *K-BILD*: King’s Brief Interstitial Lung Disease questionnaire, *CI*: Confidence interval, *FVC*: Forced vital capacity
